# Recurrent tibial intra-cortical osteosarcoma: a case report and review of the literature

**DOI:** 10.1186/1752-1947-5-93

**Published:** 2011-03-07

**Authors:** Olarn Arpornchayanon, Taninnit Leerapun, Chate Sivasomboon, Jongkolnee Settakorn, Nantawit Sugandhavesa, Dumnoensun Pruksakorn

**Affiliations:** 1 Musculoskeletal Oncology Division, Department of Orthopedics, Faculty of Medicine, Chiang Mai University, Suthep Road, Chiang Mai TH-50200, Thailand; 2 Department of Radiology, Faculty of Medicine, Chiang Mai University, Suthep Road, Chiang Mai TH-50200, Thailand; 3 Department of Pathology, Faculty of Medicine, Chiang-Mai University, Suthep Road, Chiang Mai TH-50200, Thailand

## Abstract

**Introduction:**

Intra-cortical osteosarcoma is the rarest subtype of osseous-producing tumor. Most reported cases present a low-grade histology with slow progression and good oncological control after adequate treatment. In this report, we describe a case and review the literature to propose adequate treatment.

**Case presentation:**

We present the case of a 21-year-old Thai woman who was thought to have an intra-cortical osteosarcoma of the right tibia. We performed a wide resection and reconstruction with bone transportation using an Ilizarov external fixator. The tumor recurred five years later at the same site with a similar histology. We performed a new resection and reconstruction by ankle arthrodesis with adjuvant chemotherapy. At the last follow-up, she had remained active and free from disease for seven years.

**Conclusion:**

This case report of recurrent intra-cortical osteosarcoma describes an atypical presentation. The low-grade histology, adequate surgical margin and adjuvant chemotherapy of the recurrent lesion were favorable factors, and our patient has remained free of any tumor recurrence.

## Introduction

Intra-cortical osteosarcoma is a low-grade malignancy tumor of the cortical bone which typically does not extend into the intra-medullary and surrounding soft tissues. Only 18 cases have been reported in the literature, and the most common sites are the tibia (nine cases) and the femur (nine cases). The sex ratio of females to males is 5:13, and the median age is 19 years (range, nine to 43 years). Most cases present with slow progression, and treatment results in good oncological control. However, three cases have been reported in which the patient presented with distant metastasis [1,2]. Two had local recurrences with good oncological control after *en bloc *resection [3,4]. We describe the case of a woman whose tumor was compatible with an intra-cortical osteosarcoma with a one-time recurrence. The 12-year follow-up period of this patient provides additional valuable information regarding this variant of osteosarcoma.

## Case presentation

A 21-year-old Thai woman was evaluated for a lesion in her right tibial diaphysis. The mass exhibited slow, progressive enlargement over a one-year period, and she experienced occasional pain. Her physical examination revealed a hard-consistency mass at the anteromedial aspect of the right tibia affixed to the bone. The area was mildly tender, but there was no inflammation. A bone scan showed an increased uptake of radionuclide at the site of the lesion. Her chest roentgenogram, complete blood count, blood urea nitrogen, creatinine, liver function test and serum alkaline phosphatase were within normal limits.

**Figure 1:**
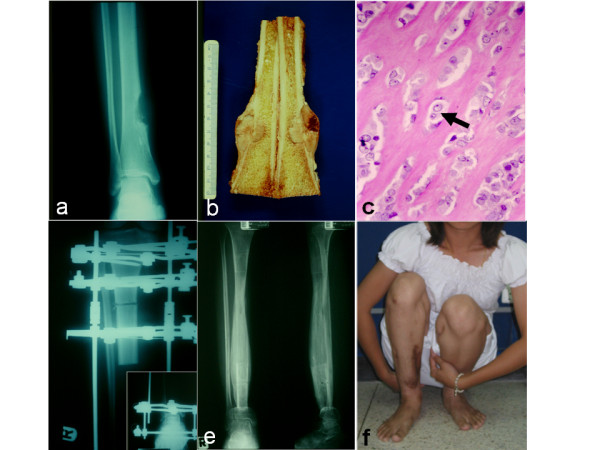
**Radiography, pathology and the treatment result in the patient's first presentation with tibial intra-cortical osteosarcoma**. **(a) **Anteroposterior radiograph of the right tibia indicating an intra-cortical lytic bone lesion. **(b) **Longitudinal section of the *en bloc *histological specimen demonstrating a well-circumscribed intra-cortical mass enclosed by periosteum. **(c) **A tissue histological specimen (first episode) shows a tumor consisting of small nests and cords of cells (arrow) surrounded by thick anastomosing branches of osteoid without any chondrosarcomatous or fibrosarcomatous matrix (hematoxylin and eosin stain; original magnification, ×400). **(d) **The tibia was reconstructed by using an Ilizarov external fixator for bone transportation. **(e) **Radiographs showing the patient's tibia six months after Ilizarov removal (two years after bone resection). **(f) **Good functional activity of the patient's knee and ankle was shown after treatment.

X-rays demonstrated an oval intra-cortical lytic bone lesion located at the diaphysis. It had an irregular endosteal border and sclerotic density that appeared to surround the osteolytic lesion. Multiple radiodense speckles were seen within the lytic area, suggesting the presence of an osteoid matrix (Figure 1a). An open incisional biopsy suggested a low-grade malignant lesion. Since surgery had been planned on the basis of X-rays, a wide margin was chosen to ensure complete excision. The patient underwent resection of the tumor (7 cm above and 3 cm below the tumor) (Figure 1b). A longitudinal section showed a 3.8 cm × 3.2 cm × 3.5 cm well-circumscribed intra-cortical mass with a 1.5 cm × 0.7 cm × 0.7 cm focus of intra-medullary extension. The tumor was grayish-white with a dull, firmly cut surface (Figure 1b). A histological section from the specimen revealed that the tumor cells had round or oval-shaped nuclei with a mild degree of nuclear atypia, a vesicular chromatin pattern and prominent nucleoli. Mitotic figures were scant (Figure 1c). No area of hemorrhage or necrosis was seen, and the surgical margin was free from malignancy tissue. The macroscopic and microscopic diagnoses were compatible with an intra-cortical osteosarcoma.

Although free fibular graft has been reported as the suitable technique for large defect reconstruction [5], in some institutions bone transportation with an Ilizarov frame currently plays an increased role in the management of large bony defects. It provides several advantages, including large bone size of new bone formation, avoidance of the risk of vascular complications, avoidance of a long period of fibular hypertrophy to obtain adequate stability and less donor site morbidity [6]. Tibial bone transportation was performed with an Ilizarov external fixator in our patient. Three rings were applied at the proximal tibia. Two rings were placed above and one was placed below the corticotomy site, and another was applied at the distal fragment. The stability of the distal part was maintained by a distal ring and an intact tibiofibular joint and distal fibular bone (Figure 1d). The bone was distracted for 450 days until the distracted fragment contacted the distal tibia (total of 14 cm long). After the ring external fixator was removed, a patellar tendon-bearing cast was applied for an additional six months to ensure the consolidation of bone (Figure 1e). The patient was finally able to walk with an almost normal range of motion of knee and ankle (Figure 1f).

**Figure 2:**
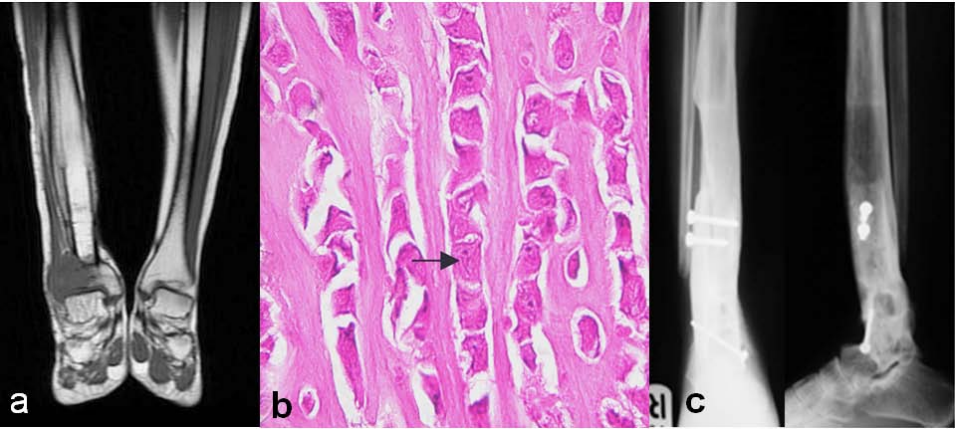
**Histological studies showing the presentation of the recurrent lesion and the result of treatment after the last operation**. **(a) **Magnetic resonance imaging scan reveals the recurrent tumor mass at the right tibial metaphysis. **(b) **A histological section of the recurrent tumor displaying nests and cords (arrow) of atypical cells, mimicking the features of the primary lesion in Figure 1c (hematoxylin and eosin stain; original magnification, ×400). **(c) **Radiographs show the final result of ankle arthrodesis six years following the second operation.

Five years later the patient felt pain in her right tibia at the same site as the primary tumor. Radiography revealed an abnormal osteolytic lesion at the lateral side of the distal tibia. Coronal T1-weighted and short-tau inversion remedy (STIR) images showed a recurrent tumor mass in the lateral portion of the right distal tibial metaphysis. The tumor showed hypointensity on T1-weighted images and mixed signal intensity on T2-weighted images with invasion into the surrounding soft tissue. Diffuse cortical thickening of the distal tibia and marrow edema was also evident (Figure 2a). A computed tomographic chest and bone scan showed no distant metastasis, and the basic laboratory findings were normal. An open biopsy indicated a low-grade malignancy (Figure 2b). A compartmental resection was performed for a slow, progressive, recurrent low-grade malignancy with limb salvage with the agreement of the patient and close long-term follow-up. The distal tibia, distal fibular joint and distal tibiofibular joint were removed 8 cm above the ankle joint, and reconstruction was performed by ankle arthrodesis using a T-plate, screws and an autologous ipsilateral strut tibial graft (Figure 2c). Neoadjuvant and adjuvant chemotherapy were administered. A review of the tumor pathology confirmed the histological finding of a low-grade osteosarcoma similar to the primary lesion with a 50% necrotic area as a result of neoadjuvant chemotherapy, and the surgical margin was free from tumor. Seven years later the patient had a painless fused ankle and was free from disease.

## Discussion

Intra-cortical osteosarcoma is the rarest variant of osteosarcoma described since the first one was reported by Jaffe in 1960 [2]. Although most cases have shown slow progression and a low-grade histological appearance, five of 18 cases have been reported as recurrent. Three of these people died as a result of distant metastasis [1,2], and two had local recurrences [3,4].

In Jaffe's original report [2], a 14-year-old boy underwent an *en bloc *excisional resection and adjuvant radiation. The patient underwent above-knee amputation, since the tumor recurred as an anaplastic spindle cell sarcoma at the same site. He died at age 26 years, five months as a result of distant metastasis. The second reported case was a 25-year-old patient with an intra-cortical osteosarcoma of the right femur. The patient underwent pre-operative cobalt radiation therapy followed by hip disarticulation and died 14 months after the diagnosis with lung metastasis. These are the only two cases in which patients underwent high-dose adjuvant radiation, and both died. Picci *et al*. [1] reported the case of patient with a recurrent intra-cortical osteosarcoma that transformed into a conventional osteosarcoma after initial treatment with local curettage. Consequently, the patient was treated with wide amputation and adjuvant chemotherapy. The lesion's potential metastasis to the lung and the patient died two years and four months after the diagnosis [1].

Lichtenstein [4] initially treated a subcortical bone lesion of the tibial shaft, erroneously diagnosed as an osteoid osteoma, by performing a local excision. The tumor, which locally recurred six months later, was diagnosed as an osteosarcoma. However, the result of the long-term follow-up was not clearly reported [4]. Scranton *et al*. [3] reported a case where a locally excised intra-cortical lesion at the femoral diaphysis resulted in multiple recurrences. Since the previous section had been re-evaluated as an osteosarcoma, the tumor was removed *en bloc*. This patient has remained asymptomatic without evidence of metastasis for 30 years [3].

Currently, several reports are in agreement that an adequate surgical margin plays an important role in controlling local recurrence [7,8]. Although surgery in our patient was considered to have been performed with an adequate margin, a 3 cm cut below the lesion in metaphysis might have been an equivocal factor leading to the secondary recurrence. The total compartment (distal tibia, distal tibiofibular joint and distal fibular joint) removal in the second episode of management with adjuvant chemotherapy was a crucial factor in disease control. The histologies of the recurrent lesions which were reported by Lichtenstein [4], Scranton *et al*. [3] and us were of low-grade malignancy, whereas Picci *et al*.'s patient [1] had a high-grade malignancy with a greater potential for metastasis. It seems that a better prognosis can be expected in recurrent lesions that present with a low-grade histology.

Among 13 well-controlled cases, eight were treated without adjuvant chemotherapy and were free from recurrence and metastasis for 21 months (range, seven to 141 months). Five patients received adjuvant chemotherapy and remained well without recurrence and metastasis for 48 months (range, 10 to 84 months). Our patient was treated without adjuvant chemotherapy at the first presentation because her tumor was considered to be a histologically low-grade lesion and an adequate margin had been used during surgery. We eventually had to combine chemotherapy with surgery during the second episode to minimize the risk of recurrence.

## Conclusion

The ideal treatment of intra-cortical osteosarcomas is surgical resection with an adequate margin. In cases of uncertain margin resection, adjuvant chemotherapy should be considered. For a recurrent lesion, low-grade histology is a favorable prognostic factor. However, adequate margin resection as well as neoadjuvant and adjuvant chemotherapy must be proposed in cases of lesion recurrence to offer the patient a good prognosis.

## Consent

Written informed consent was obtained from the patient for publication of this case report and any accompanying images. A copy of the written consent is available for review by the Editor-in-Chief of this journal.

## Competing interests

The authors declare that they have no competing interests.

## Authors' contributions

DP wrote the draft manuscript. OA, TL, NS and DP carried out the operation and the patient follow-up. CS participated in the radiological evaluation. JS participated in the pathological studies. All authors read and approved the final manuscript.
